# Optimizing C-Type Natriuretic Peptide and Receptor Expression Analysis with Droplet Digital™ PCR: Advancing Biomarker Discovery for Brugada Syndrome?

**DOI:** 10.3390/biom15060792

**Published:** 2025-05-29

**Authors:** Manuela Cabiati, Federico Vozzi, Elisa Persiani, Marcello Piacenti, Andrea Rossi, Agnese Sgalippa, Antonella Cecchettini, Gianluca Solarino, Giulio Zucchelli, Lorenzo Mazzocchetti, Pasquale Notarstefano, Letizia Guiducci, Maria Aurora Morales, Silvia Del Ry

**Affiliations:** 1 Laboratory of Biochemistry and Molecular Biology, CNR Institute of Clinical Physiology, Via Giuseppe Moruzzi 1, 56124 Pisa, Italy; manuela.cabiati@cnr.it (M.C.); federico.vozzi@cnr.it (F.V.); elisa.persiani@cnr.it (E.P.); agnese.sgalippa@santannapisa.it (A.S.); antonella.cecchettini@unipi.it (A.C.); letizia.guiducci@cnr.it (L.G.); auroramorales@icloud.com (M.A.M.); 2 Fondazione Toscana Gabriele Monasterio, 56124 Pisa, Italy; marcello.piacenti@ftgm.it (M.P.); andrea.rossi@ftgm.it (A.R.); 3 Health Science Interdisciplinary Center, Sant’Anna School of Advanced Studies, 56100 Pisa, Italy; 4 Department of Clinical and Experimental Medicine, University of Pisa, 56126 Pisa, Italy; 5 University Cardiology Division, Versilia Hospital, 55041 Lido di Camaiore, Italy; gianluca.solarino@uslnordovest.toscana.it; 6 Second Division of Cardiology, Azienda Ospedaliero Universitaria Pisana, 56126 Pisa, Italy; zucchelli76@gmail.com (G.Z.); info@aritmologomazzocchetti.it (L.M.); 7 Cardiovascular and Neurological Department, San Donato Hospital, 52100 Arezzo, Italy; pasqualenotarstefano@gmail.com

**Keywords:** C-type natriuretic peptide, mRNA, *NPR-B*, *NPR-C*, Brugada syndrome, droplet digital™ PCR

## Abstract

Brugada syndrome (BrS) is a cardiac disease associated with characteristic ECG abnormalities and a heightened risk of sudden cardiac death, especially in young individuals with structurally normal hearts. The primary aim of this study was to highlight, for the first time, the potential of using droplet digital PCR (ddPCR), a highly sensitive method, to detect *C-type natriuretic peptide* (*CNP*) and its receptors, *NPR-B* and *NPR-C*, expression in BrS. Whole-blood samples from 12 subjects with type 1 BrS and 12 controls were analyzed. *CNP* expression was detectable and lower in BrS patients than in the controls, although not significantly. *NPR-B* and *NPR-C* expression was significantly reduced in the same patients (*p* ≤ 0.05). Strong correlations were observed between *CNP* and *NPR-B* (*p* = 0.01) and *NPR-C* (*p* < 0.0001), as well as between *NPR-B* and *NPR-C* (*p* = 0.0002). Body weight correlated with *CNP* (*p* = 0.02), *NPR-B* (*p* = 0.03), and *NPR-C* (*p* = 0.02); meanwhile, *NPR-B* expression was related to height (*p* = 0.05). This study is the first to analyze *CNP* expression and its specific receptors using ddPCR technology, showing for the first time their presence and activation in individuals with BrS. Although further research is needed to clarify CNP-related mechanisms, these findings offer a valuable starting point for exploring its role in BrS.

## 1. Introduction

The growing understanding of genome structure and variation has led to the development of advanced technologies that enable researchers to simultaneously study thousands of genes, transcripts, and proteins. However, despite these advances, many diseases remain poorly understood. This highlights a significant challenge in biomedical research: the urgent need for more sophisticated techniques to identify potentially involved genes, especially in complex or rare conditions where current methods fall short.

Brugada syndrome (BrS) is a clinical condition first identified in 1992, marked by characteristic baseline ECG abnormalities and an increased risk of sudden cardiac death, particularly in young patients with no evident structural heart abnormalities. So far, three ECG types have been described. The hallmark feature of type 1 BrS is represented by a coved-type ST elevation in the right precordial leads (V1–V3) on the baseline ECG, often accompanied by episodes of ventricular tachyarrhythmias [1,2]. The prevalence of the syndrome is estimated at around 15 per 10,000 individuals in Southeast Asia, including Japan, and 2 per 10,000 in Western countries. BrS is attributed to 4–12% of all sudden deaths and up to 20% of sudden deaths in individuals with structurally normal hearts. It is 8 to 10 times more common in men than in women [3].

One of the main challenges remains the lack of a precise diagnostic cut-off, and the distinction between specific ECG patterns is not always easy to determine and is often influenced by clinicians’ interpretations. As observed, even though specific patterns are clearly described in the scientific literature, individual ECGs frequently show variations, complicating their interpretation and making diagnosis uncertain [4].

Genetic studies have identified mutations in the SCN5A gene, which encodes the alpha subunit of the cardiac sodium channel, as a primary cause of BrS, accounting for approximately 20–30% of cases. These mutations reduced sodium current, disrupting transmembrane ion flux during the cardiac action potential and increasing susceptibility to arrhythmias [2]. While various variants in the SCN5A gene have been identified, the pathogenic role of each variant remains to be experimentally validated [5]. A significant limitation is that variants have been explored using animal models or heterologous expression systems, which may not fully represent the cardiac physiological conditions. Recent data suggest that genetics are linked with the severity of cellular and clinical phenotypes, as observed in BrS patients carrying SCN5A mutations who undergo electrophysiology studies and epicardial mapping [6,7].

Although advancements in understanding BrS have been made, the underlying pathogenic mechanism remains unclear. Currently, genetic screening in BrS serves only to identify potential mutations and does not impact prognosis or treatment decisions [8].

Recent research has emphasized the significant involvement of inflammation in the pathogenesis of BrS [9,10]. A case report study illustrated that two BrS patients with acute cardiac inflammation encountered frequent episodes of ventricular fibrillation, suggesting a potential link between inflammation and the occurrence of malignant BrS ventricular arrhythmias [11]. Nevertheless, the precise roles and pathological mechanisms of inflammation in BrS remain ambiguous.

A valuable source of information for understanding the mechanisms underlying BrS could be the analysis of specific circulating biomarkers using an innovative approach applicable in precision medicine, such as transcriptomic analysis. In the past decade, transcriptomics has significantly advanced cardiovascular biomarker research, with several now approved for clinical use [12].

The rarity of BrS, combined with variable ECG interpretation and the absence of a definitive biomolecular marker, limits its recognition and increases the risk of severe outcomes.

For this reason, a new paradigm is needed in BrS diagnosis, integrating traditional clinical guidelines with innovative approaches to develop diagnostic strategies within the framework of cardiovascular precision medicine.

The inflammatory circulating biomarkers may help to close this gap as inflammation can alter cardiac electrophysiology and structure.

Among them all, *C-type natriuretic peptide* (*CNP*) could represent a valuable potential biomarker due to its direct action at the cardiac level and presenting anti-inflammatory activities [13]. *CNP* belongs to the natriuretic peptide (*NP*) family [14,15,16,17] and exerts its biological activity primarily through binding to its specific receptor, *NPR-B*, which stimulates guanylate cyclase. This interaction increases intracellular levels of the second messenger cyclic guanosine monophosphate (cGMP), thereby contributing to the regulation of several physiological processes [18].

Subsequently, cGMP activates protein kinases G I and II, which modulate various cellular functions through the phosphorylation of specific target proteins [19].

In addition to *NPR-B*, *CNP* also binds to *NPR-C* [19].

Although *NPR-C* was initially regarded solely as a clearance receptor lacking signaling activity, it was later found to possess pertussis toxin-sensitive Gi-binding domains within its intracellular C-terminal tail. These domains facilitate the inhibition of adenylyl cyclase and the activation of phospholipase C-β [20].

Previous research indicates that *CNP* also reduces neuronal calcium currents and intracellular calcium transients induced by depolarization in isolated stellate sympathetic neurons [21]. A recent study [22] showed that intravenous application of *CNP* could significantly inhibit the activation of the cardiac sympathetic nervous system and stabilize cardiac electrical activity through the cGMP signaling pathway, thereby playing a protective role in ventricular arrhythmias after myocardial ischemia.

While *NP*s have long been recognized for their role in regulating fluid balance and blood volume, recent findings highlight their critical involvement in cardiac electrophysiology and the development of arrhythmias. This is supported by multiple studies showing the influence of *NP*s on the ion channel activity and electrical properties across various cardiac cell types and regions. Moreover, mutations in components of the *NP* system have been linked to atrial fibrillation in humans, underscoring their functional importance. Nonetheless, despite the robust evidence for their role in modulating cardiac electrical function, reported effects of NPs have been inconsistent. These discrepancies may reflect the intrinsic complexity of *NP* signaling, which involves multiple receptors and downstream pathways that may be selectively engaged depending on the cellular context or physiological state [22,23].

The primary aim of this study was to highlight the potential of using a novel methodology, droplet digital™ PCR (ddPCR), capable of detecting the expression of biomarkers that cannot be identified with less sophisticated techniques, such as Real-time PCR. To this end, potential fluctuations in the expression levels of *CNP* and its specific receptors, *NPR-B* and *NPR-C*, were evaluated in individuals with type 1 BrS. The ddPCR offers the advantage of direct and absolute quantification of the target genes without the need for standard curves, providing more precise and reproducible data compared to Real-time PCR [24,25,26,27]. Furthermore, its end-point measurement approach allows nucleic acid quantification regardless of reaction efficiency. This allows for clear positive or negative identification of each droplet and supports multiplex detection of target molecules [28]. As a result, ddPCR technology can quantify extremely low-abundance biomarkers [24,25,26,27,28]. Additionally, liquid biopsies samples analyzed using ddPCR do not require normalization with housekeeping genes as they account for the initial volume of blood used in the analysis.

Currently, no studies in the literature have analyzed *CNP* and its specific receptors using ddPCR, neither with primers nor with probes. Therefore, this study could represent a starting point for identifying the most appropriate approach to study *CNP* and its receptors using this advanced and highly sensitive technology. By analyzing whole-blood samples from individuals with and without BrS, this research can provide new insights into the role of *CNP* in the pathophysiology of the syndrome.

## 2. Materials and Methods

### 2.1. Subjects Enrollment and Plasma Collection

This is a multicenter, non-randomized, retrospective, and non-profit study. The investigation conforms to the principles outlined in the Declaration of Helsinki. The local ethics committee “Comitato Etico Regionale per la Sperimentazione Clinica della Regione Toscana Sezione: AREA VASTA NORD OVEST” approved the study, and informed consent was obtained from each subject’s parents [29].

The type 1 BrS patients were enrolled by investigators from hospitals in central Italy (Cardiology Division, Versilia Hospital, Lido di Camaiore; Second Division of Cardiology, Azienda Ospedaliero Universitaria Pisana, Pisa; Cardiovascular and Neurological Department, San Donato Hospital, Arezzo; Fondazione Toscana Gabriele Monasterio, Pisa).

The ECG diagnosis of BrS adhered strictly to the guidelines outlined in the 2015 European Society of Cardiology recommendations for managing patients with ventricular arrhythmias and preventing sudden cardiac death [3]. Inclusion criteria for enrolling patients included the presence of type 1 BrS ECG pattern electrocardiographic changes, or in cases of high clinical suspicion (such as familial history of BrS or surviving cardiac arrest without an apparent cause), age between 14 and 65 years [3,29,30]. The control group consisted of individuals of similar age undergoing outpatient cardiological examinations, showing a standard resting ECG. Exclusion criteria included the presence of structural cardiac disease, concurrent conditions that may interfere with protocol completion, lack of informed consent, pregnancy, history of coronary artery disease, and severe renal or hepatic insufficiency. Structural heart disease was ruled out in all patients before enrollment through non-invasive imaging techniques (echocardiography and/or cardiac MRI).

We studied 24 subjects, including 12 controls (C) and 12 subjects with spontaneous type 1 BrS. Both BrS patients and controls underwent baseline ECGs using standard recording parameters (paper speed 25 mm/s, amplification 10 mm/mV, and a sampling rate of 10 s at 500 Hz).

For molecular biology studies, whole-blood samples were gathered in PAXgene blood RNA system tubes (DIALAB ITALIA Srl, Milan, Italy), which include reagents to stabilize RNA immediately. This technique effectively preserves intracellular RNA, allows the sample to be stored at temperatures between −20 and −80 °C, and ensures that the sample maintains the same purity level as fresh blood.

### 2.2. RNA Extraction, Reverse Transcription, and Droplet Digital™ PCR Workflow

Total RNA was isolated from samples collected in PAXgene tubes using a specific kit (PAXgene Blood RNA Kit, Qiagen, Milan, Italy), as previously described [31,32]. The total RNA sample concentration was determined by measuring the absorbance at 260 and 280 nm (NanoDrop Thermofisher, Waltham, MA, USA) and calculated using the Beer–Lambert law (expected values between 1.8 and 2.1). The reading ratio at 260 nm and 280 nm (A260/A280) provides an estimate of RNA purity with respect to contaminants absorbing in the UV spectrum, such as protein. Samples showing OD 260/280 ratios of 1.8–2.1 were used. After integrity, purity, and concentration evaluation, the RNA samples were stored at −80 °C.

Approximately 0.5 μg of total RNA from each sample was reverse transcribed with the iScript cDNA synthesis kit (Bio-Rad, Hercules, CA, USA) according to the manufacturer’s instructions.

#### *CNP*, *NPR-B*, and *NPR-C* Expression Analysis by ddPCR

In ddPCR, RNA is retrotranscribed into cDNA and then amplified in thousands of individual reaction chambers, created by a water-in-oil emulsion that divides the sample into approximately 20,000 droplets. PCR amplification occurs within each droplet, and the target molecule’s compartments are identified as positive (using specific dyes or fluorescent probes). Droplets without the target molecule are considered negative. To reliably assess the absolute number of mRNA copies for the gene of interest in ddPCR, a minimum of about 15,000 droplets is required, based on the Poisson distribution. A general overview of the ddPCR method is provided in the paper by Galimberti and colleagues [33], but no specific guidelines for ddPCR settings are available at present. However, as this technique becomes increasingly widespread in various laboratories, the scientific community has produced two valuable resources to ensure high-quality assays, the ISO 20395:2019 standards [34]. (available at https://www.iso.org/obp/ui#iso:std:iso:20395:ed-1:v1:en, accessed on 28 February 2022) and the dMIQE guidelines, which summarize the essential information for reporting ddPCR experiments [35].

For *CNP* expression, the cDNA diluted 1:2 (12.5 ng/μL) was added to ddPCR Eva Green Supermix (Bio-Rad Laboratories, Hercules, CA, USA) along with *CNP* pre-cust primers (Hs_NPPC_2_SGQuantitect Primer Assay, QIAGEN, Milan, Italy). The positive and negative droplets were measured using a QX200™ droplet reader (Bio-Rad Laboratories, CA, USA). The absolute copy count was calculated based on the droplet count using the Poisson distribution and the Quanta Soft software 1.7.4.0917 (Bio-Rad Laboratories, CA, USA).

During the *CNP* ddPCR set-up phase, a cDNA pool from PAXgene whole-blood samples (Pool) was used. cDNA of human induced pluripotent stem cells-derived cardiomyocytes (hiPSC-derived CMs) was used as a positive control (hiPSC-CM). The experiment was performed at three different temperatures (Ta = 55 °C, 58 °C, 60 °C).

For *NPR-B* and *NPR-C*, ddPCR multiplexing was used. This multiplex assay relies on the amplitude of the amplifiers. Different targets are identified by probes labeled with the same fluorophore (FAM or HEX), but at different concentrations. This strategy quantifies up to four targets within a single reaction.

In particular, the final reaction volume was 22 μL, using 5 μL of cDNA (12.5 ng/μL), 11 μL of the 2XddPCR Supermix for probes (no dUTP) (Bio-Rad, Hercules, CA, USA), probe gene expression for *NPR-B* (1.1 μL, dye quencher: 5′-6-FAM, 3′Iowa Black TM FQ, code dHsaCPE5039024) and NPR-C (0.7 μL, dye quencher: 5′HEX, 3′Iowa Black TM FQ, code dHsaCPE5034833), and 4.2 μL of H_2_O. Following the manufacturer’s guidelines, 70 µL of droplet generation oil for probes was added. The water-in-oil droplet emulsion was created using the QX200 droplet generator (Bio-Rad, Hercules, CA, USA). The amplification step was carried out with 40 µL of emulsion in a C1000 thermal cycler (Bio-Rad, Hercules, CA, USA) using the following outlined program: probe ddPCR-polymerase activation at 95 °C for 10 min, 40 cycles of amplification at 94 °C for 30 sec (denaturation) and 55 °C for 1 min (annealing), droplets stabilization at 98 °C for 10 min, followed by an infinite hold at 4 °C. A ramp rate of 2 °C/s was used among the steps of the amplification. After amplification, positive and negative droplets were read using a QX200 droplet reader (Bio-Rad, Hercules, CA, USA).

As for the *NPR-B* and *NPR-C* set-up, hiPSC-derived CMs cDNA were used as a positive control.

### 2.3. Statistics

In the ddPCR method, the expression values were obtained as copy/μL for each sample using the QXManager software version 2.1 (Bio-Rad, Hercules, CA, USA) for the absolute quantification of the target genes, and were reported as copy/2.5 mL of whole blood. Regarding data pre-processing, we ensured the quality control of ddPCR data by using essential positive/negative controls to ensure the reliability of the results and the proper functioning of the ddPCR reaction.

Statistical analysis was performed using StatView 5.0.1 software released by Windows Statistical (SAS Institute, Inc., Cary, NC, USA). Skewed variables were log-transformed before statistical analysis. The unpaired Student t-test assessed differences between two independent groups; moreover, Fisher’s test was used to assess differences between more than two independent groups after an ANOVA, whilst the relations between variables were evaluated using linear or multivariate logistic regression.

To address confounding factors, we performed multivariate regression analysis between the independent variables (age, BMI, diastolic and systolic pressure, weight, height, and heart rate) and the outcome. Results are presented as mean ± S.E.M. A *p*-value < 0.05 was considered statistically significant.

## 3. Results

### 3.1. BrS Patient’s Characterization

**Figure 1:**
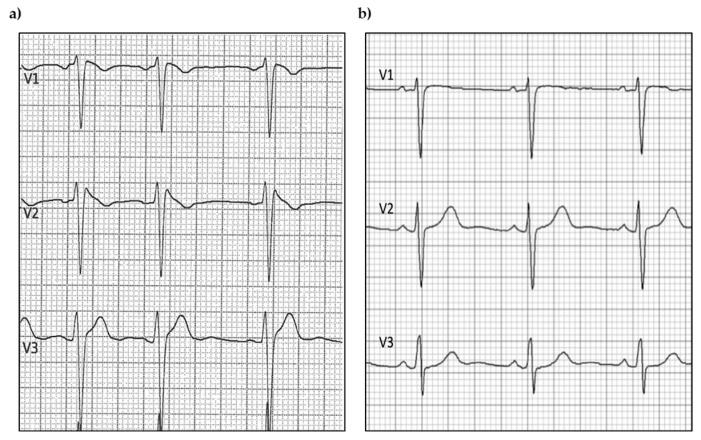
Example of (**a**) BrS and (**b**) control ECG traces.

BrS patients showed prominent coved ST-segment elevation with a J wave amplitude or ST-segment elevation > 2 mm at its peak, followed by a negative T-wave, with little or no isoelectric separation in the right precordial leads (V1 to V3). Figure 1 depicts ECG traces derived from a BrS patient and a control, demonstrating the significant differences between the two groups’ baseline ECGs.

Table 1 reports the clinical and auxologic characteristics of both BrS and C.

### 3.2. Biomolecular Analysis: Droplet Digital PCR Results

**Figure 2:**
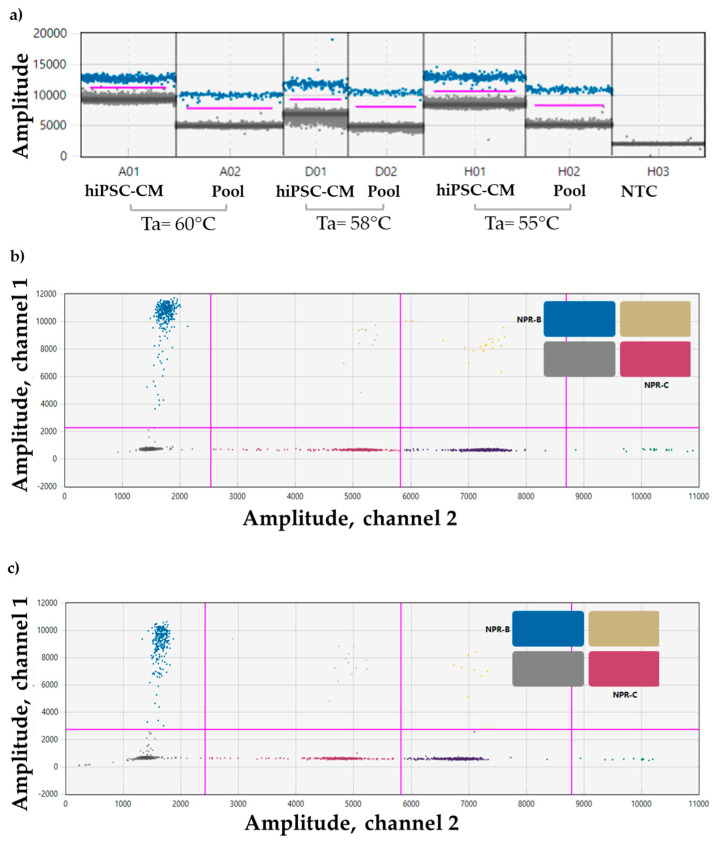
Set-up of a ddPCR for (**a**) *CNP* primer in hiPSC-CM (positive control), and in the Pool at 3 different Tas (55 °C, 58 °C, and 60 °C); sample dilution 1:2. Event count mean of about 13,000. (**b**) *NPR-B* (blue droplet population) and *NPR-C* (purple droplet population) probes in the Pool, and in (**c**) hiPSC-CM (positive control); sample dilution 1:2. Event count mean of about 14,000. Ta = 55 °C. ddPCR droplet clouds located on the right section of the 2D plot are representative of other genes not included in the manuscripts (yellow, dark violet, orange, and green dots).

The *CNP* expression was confirmed in the Pool, and the optimal Ta resulted to be 55 °C (Figure 2a).

Also, multiplex experiments for *NPR-B* and *NPR-C* confirmed their expression in the Pool (Figure 2b) and in the hiPSC-CM used as positive control (Figure 2c).

When analyzing control and BrS patients, *CNP*, *NPR-B*, and *NPR-C* expression were detected.

**Figure 3:**
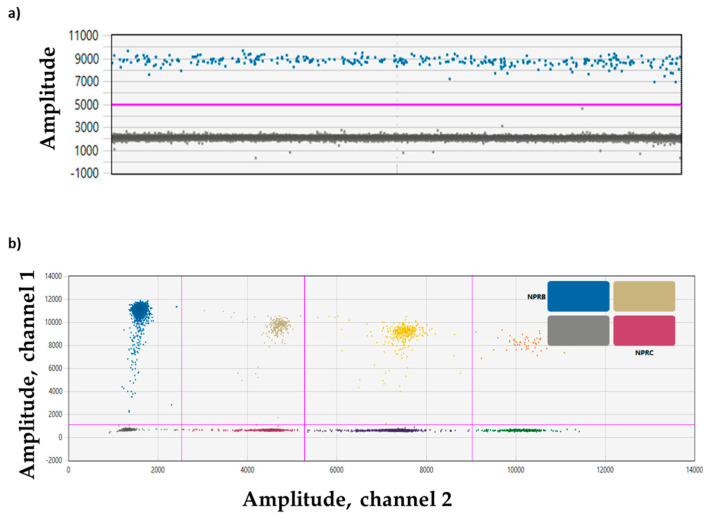
Plots of droplet fluorescence. (**a**) ddPCR data from a singleplex experiment with a single target (*CNP*) detected per channel and viewed as a 1D plot with each droplet from a sample plotted on the graph of fluorescence intensity vs. droplet number. All positive droplets (*CNP*) are above the red threshold line (blue) while all negative droplets (gray) are below the red threshold line. (**b**) ddPCR data from a multiplex experiment in which *NPR-B* and *NPR-C* targets are amplified and viewed in a 2D plot. Channel 1 fluorescence (FAM) is plotted against channel 2 fluorescence (HEX) for each droplet. Each dot on the figure represents one droplet containing at least one copy of the target: *NPR-B* (blue), *NPR-C* (violet), and *NPR-B*+*NPR-C* (double-positive FAM/HEX droplets, beige). All negative droplets (double-negative FAM/HEX droplets) are in gray. ddPCR droplet clouds located on the right section of the 2D plot are representative of other genes not included in the manuscripts (yellow, dark violet, orange, and green dots).

Figure 3 shows an example of a singleplex experiment for the *CNP* target (Figure 3a) and a multiplex experiment for *NPR-B* and *NPR-C* (Figure 3b) in a BrS patient.

**Figure 4:**
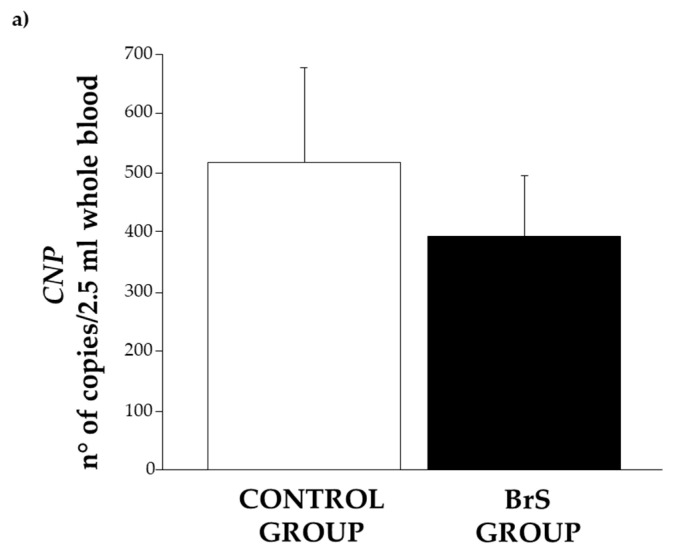

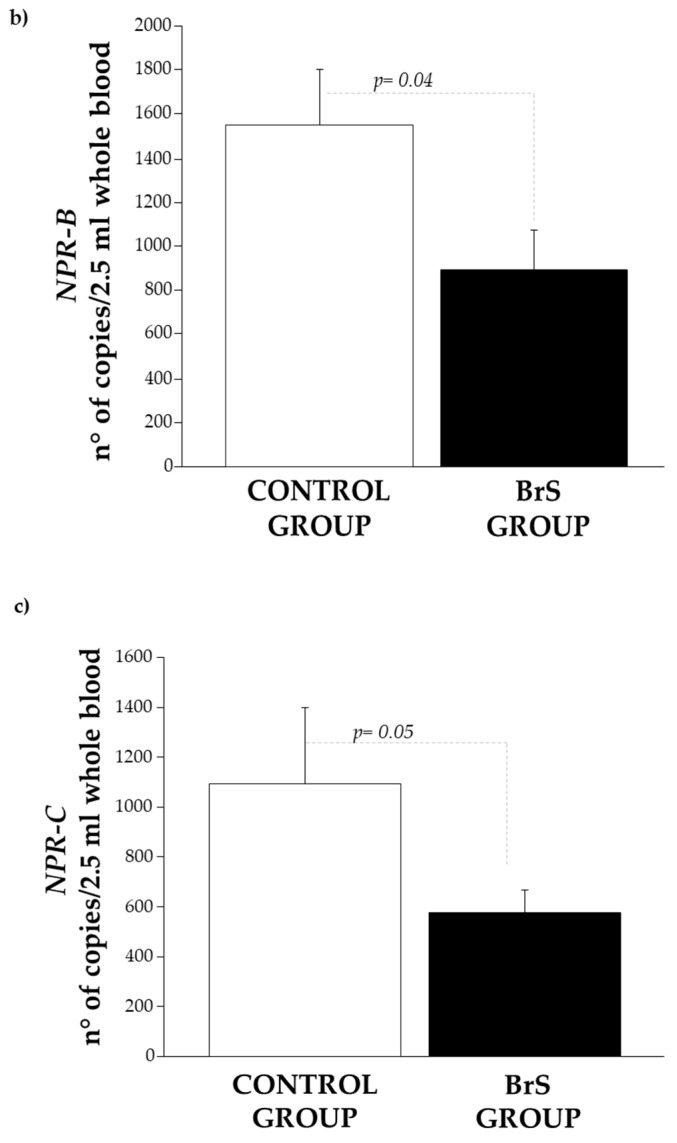
Expression level of (**a**) *CNP*, (**b**) *NPR-B*, and (**c**) *NPR-C* in the control group (white bar) and BrS group (black bar). Data are expressed as mean ± SEM (error bars) for each group.

In patients with BrS, the expression of *CNP* was observed to be lower than in the control group. However, the difference did not reach statistical significance (Figure 4a).

Similarly, its specific receptors, *NPR-B* (Figure 4b) and *NPR-C* (Figure 4c), were also significantly reduced (*p* = 0.04 and *p* = 0.05, respectively).

**Figure 5:**
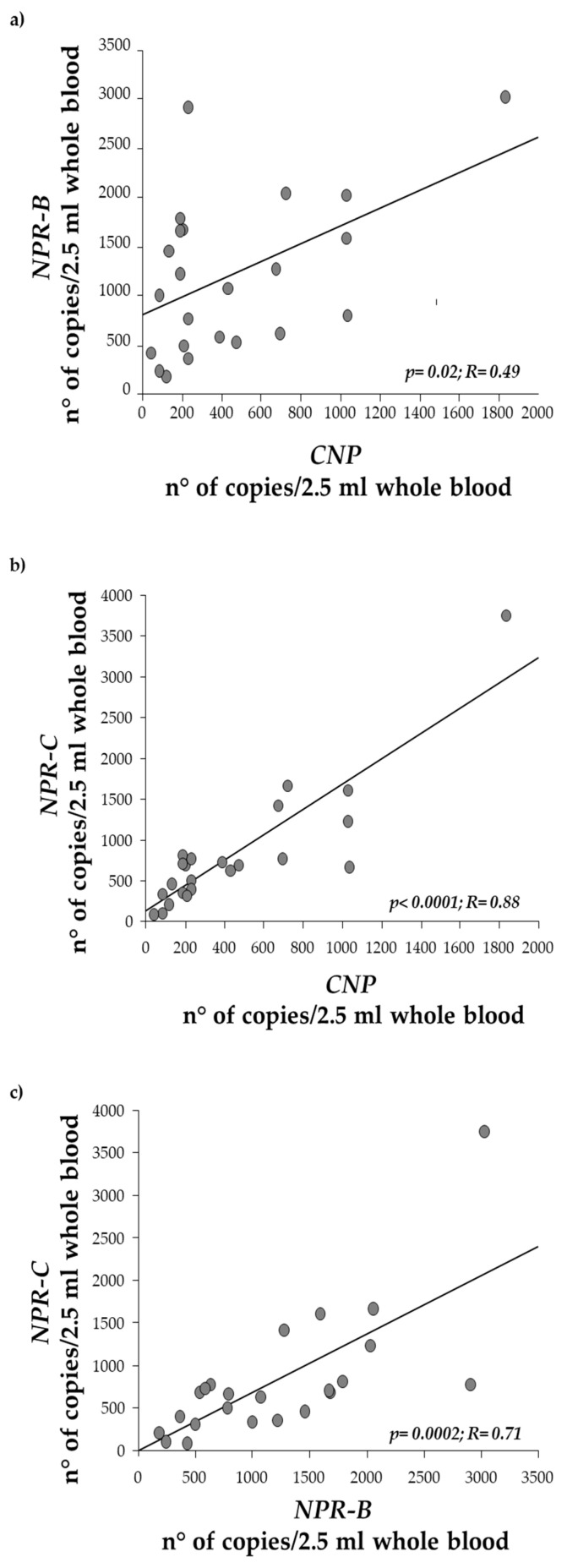
Regression analysis between *CNP* expression levels and (**a**) *NPR-B*, (**b**) *NPR-C*, and (**c**) between *NPR-B* and *NPR-C*.

We also identified a robust and statistically significant correlation between *CNP* and *NPR-B* (Figure 5a, *p* = 0.01), as well as between *CNP* and *NPR-C* (Figure 5b, *p* < 0.0001). A strong correlation was also observed between *NPR-B* and *NPR-C* (Figure 5c, *p* = 0.0002).

We also observed a correlation between body weight, *CNP* levels, and its specific receptors *NPR-B*/*NPR-C*, as reported in Table 2, as well as between BMI and *CNP*, *NPR-B*, and *NPR-C*, respectively, in both the control group and BrS patients (Table 2). A significant relationship was also found between *NPR-B* expression and height (Table 2).

The multivariate regression analysis did not show a statistically significant association between the independent variables (age, BMI, diastolic and systolic pressure, weight, height, and heart rate) and the outcome.

Given that some patients in the BrS group were hypertensive and exhibited higher BMI values compared to the control group, we wish to emphasize that the BMI of BrS patients remained within the normal or slightly overweight range. This degree of variation is unlikely to account for differences in *CNP* pathway expression. Moreover, evidence indicates that dysregulation of the *CNP*–*NPR-B* signaling pathway is associated with hypertension, tachycardia, and impaired left ventricular systolic function, primarily through mechanisms involving increased sympathetic activity. These alterations may represent early indicators in clinically asymptomatic individuals who harbor latent dysfunctions that could be unmasked by external stressors.

Importantly, when we reanalyzed the data excluding hypertensive subjects from both groups no changes were observed in the expression levels of the analyzed biomarkers, either in terms of trend or statistical significance (*CNP*: 516.4 ± 160.19 vs. 201.09 ± 51.6 n° copies/2.5 mL whole blood, *p* = ns; *NPR-B*: 1546.8 ± 258.97 vs. 724.71 ± 213.5 n° copies/2.5 mL whole blood, *p* = 0.04; *NPR-C*: 1181.045 ± 325.31 vs. 396.91 ± 103.2 n° copies/2.5 mL whole blood, *p* = 0.01). This further supports the hypothesis that the observed alterations are independent of hypertensive status and BMI variations and may be directly linked to BrS pathophysiology.

## 4. Discussion

This study is the first that uses the droplet digital^TM^ PCR technology to identify *CNP* system expression. This new technology provides an innovative approach for directly quantifying target genes, offering greater precision and reproducibility than Real-time PCR [33]. This advantage stems from ddPCR’s ability to partition the sample into thousands of individual droplets, allowing for absolute quantification of nucleic acids without the need for standard curves. This method minimizes the impact of inhibitors and variability in amplification efficiency, making it especially useful when working with complex samples, where traditional Real-time PCR may yield less reliable results. This study is the first to analyze *CNP* expression and its specific receptors using ddPCR technology, showing for the first time their presence and activation in individuals with Brugada syndrome and offering insights into the potential role of the *CNP* system in BrS.

The lower expression of *CNP* in BrS patients suggests a possible trend toward down-regulation of this peptide, even if it is not statistically significant. The more substantial and statistically significant reduction in *NPR-B* expression can indicate that an impaired receptor-mediated signaling contributes to BrS. The significant decrease in *NPR-C* mRNA levels may indicate the involvement of distinct regulatory mechanisms or suggest that *NPR-C* plays a different, yet currently unidentified, role in BrS.

The correlations between *CNP* and its receptors (*NPR-B* and *NPR-C*) suggest a coordinated regulation of the *CNP* signaling pathway.

The robust correlation between *NPR-B* and *NPR-C* also points to a possible interplay between these receptors in modulating *CNP*’s effects. Given the role of *CNP* in cardiovascular homeostasis [36], including vasodilation and antifibrotic effects [36,37,38], these correlations indicate that any dysregulation of one component (*CNP* or its receptors) could disrupt the overall balance of signaling, potentially contributing to the arrhythmic characteristics of BrS.

*CNP* is a hormone that functions in a paracrine manner. Within the cardiovascular system, CNP produced by endothelial cells enhances the effects of endocrine cardiac hormones like atrial natriuretic peptide (*ANP*) and B-type natriuretic peptide (*BNP*), primarily by regulating vascular tone and maintaining the integrity of the endothelial barrier [16,38]. *CNP* is mainly found in coronary endothelial cells in the heart, although it is also present in smaller amounts in cardiomyocytes and fibroblasts [39,40]. Its levels rise in both clinical and experimental heart disease [40,41]. The administration of synthetic *CNP* or stabilized peptide mimetics has shown protective effects against hypertrophy and fibrosis in preclinical models of pathological cardiac remodeling, such as those involving experimentally induced myocardial infarction [41,42]. Additionally, mice with targeted deletion of the *CNP* gene in cardiomyocytes or fibroblasts exhibited increased cardiac hypertrophy and fibrosis in response to pressure overload in the left ventricle [42].

In our study, the multivariate regression analysis did not show a statistically significant association between the independent variables (age, BMI, diastolic and systolic pressure, weight, height, and heart rate) and the outcome. This suggests that the factors considered do not appear to influence the expression of the analyzed genes, as also confirmed by the results obtained after excluding hypertensive patients from the analysis.

While the role of *CNP* and its receptors in BrS remains preliminary and not yet applicable for clinical use, our findings may provide a valuable starting point for future research. Future studies with larger sample sizes and an analysis of additional variables may provide a clearer understanding of the role of these factors.

As reported above in BrS, the hallmark feature is manifested as a coved-type ST elevation in the right precordial leads (V1–V3) on the ECG: episodes of ventricular tachyarrhythmias up to sudden death may also occur [1,2]. It is known [42] that *CNP* reduces cardiac sympathetic neurotransmission via a reduction in neuronal calcium signaling and norepinephrine release through the NPR-B receptor.

*CNP*, like the other *NP*s, also plays a crucial role in controlling heart rate and modulating the electrophysiological characteristics of the heart, primarily through its influence on ion channels in both cardiomyocytes and cardiac fibroblasts. This regulatory function is further supported by the discovery of mutations within the *NP* signaling system that are directly linked to cardiac arrhythmias, including atrial fibrillation. Collectively, these findings highlight the fundamental importance of *NP*s in maintaining normal cardiac electrical activity and in the pathogenesis of arrhythmogenic disorders [22].

Moreover, the disruptions in the *CNP*/*NPR-B* signaling pathway have been linked to hypertension, tachycardia, and impaired left ventricular systolic function due to sympatho-excitation. These parameters could serve as key indicators in individuals who appear clinically healthy but may have an underlying pathology that could be triggered by external factors. Based on our results, *CNP* may be considered a promising candidate due to its potential involvement in both inflammatory processes and cardiac electrical disturbances. Moreover, the use of ddPCR technology provides an additional tool by quantifying extremely low target levels, thereby enabling the detection of biomarker activity that might otherwise go unnoticed with other methodologies. In syndromes like this, where established indicators are absent, even a minimal variation in a biomarker can have significant implications.

## 5. Limitations, Future Directions and Conclusions

The findings of this work provide a foundation for further investigation, suggesting that the *CNP* system could contribute to BrS pathophysiology. The main limitation of this study is the small sample size, which is also a challenge due to the nature of the syndrome being studied which makes it difficult to enroll a large number of patients.

Regarding the main aim of the study, which involved the detection of low-abundance transcripts by ddPCR, the goal was achieved.

Furthermore, in our study, although a complete stratification based on all clinical aspects of BrS was not available, we included several relevant clinical variables that provide a useful context for interpreting gene expression data, reflecting, albeit partially, the clinical heterogeneity of the cohort analyzed.

The absence of more detailed phenotypic data represents a further limitation of the study and may reduce the ability to establish more precise correlations with the observed molecular alterations. For this reason, we emphasize the need for future studies that incorporate broader clinical phenotyping to better understand the link between transcriptomic profiles and clinical manifestations of BrS.

Taking into account the limited number of patients, preclinical models replicating the human pathophysiology of BrS have become crucial for advancing our understanding of the disease and developing effective strategies for its prevention, diagnosis, and clinical management. Various approaches, including in vivo animal studies, in vitro cellular systems, and in silico computational models, have been employed to investigate BrS, each with distinct advantages and limitations.

Animal models, particularly rodents, provide the opportunity to study BrS in a living, dynamic system that can be humanized through transgenesis. However, significant differences in anatomy and electrophysiology between animal models and humans pose challenges in fully replicating human arrhythmias.

Cellular models, especially those derived from induced pluripotent stem cells (iPSCs), offer a more physiologically relevant alternative. Advances in differentiation protocols have enabled the generation of nodal, atrial, and ventricular cardiomyocytes, providing a powerful platform for disease modeling. However, current cellular models do not fully capture the subcellular and electrophysiological complexities of BrS, highlighting the need for improved differentiation strategies and more sophisticated in vitro systems.

Computational modeling of BrS remains particularly challenging due to its complex and largely unknown genetic substrate, which makes it difficult to accurately reproduce defective ion currents. However, in silico models hold great potential for clinical risk stratification, helping to predict fatal arrhythmic events in diagnosed patients and refining personalized treatment approaches.

## Figures and Tables

**Table 1:** Clinical and auxologic characteristics of patients with Brugada syndrome.

	CONTROLS	BrS PATIENTS	*p*
ANTHROPOMETRIC VARIABLES			
Sex, Male	6/12	10/12	ns
Age, years	40.2 ± 4.1	49.2 ± 3.8	ns
Smoke	2/12	3/12	ns
Sex, Male	6/12	10/12	ns
METABOLIC VARIABLES			
Type 2 diabetes mellitus	0/12	1/12	ns
Dyslipidemia	2/12	2/12	ns
Weight, kg	68.1 ± 4.5	75.0 ± 5.0	ns
Height, cm	171.4 ± 3.3	170.0 ± 3.4	ns
Body Mass Index	22.9 ± 0.7	25.8 ± 0.8	0.03
CLINICAL VARIABLES			
Sudden death familiarity	3/12	3/12	ns
Family history of BrS	0/12	0/12	ns
Positive SCN5A gene mutation	0/12	3/12	ns
Pre-syncope episodes	1/12	2/12	ns
Hypertension	0/12	5/12	0.001
Heart rate	62.3 ± 3.6	70.0 ± 8.6	ns
Implantable cardioverter-defibrillator	0/12	3/12	ns
Tachycardia	8/12	2/12	0.05
Ventricular extrasystole	5/12	0/12	0.006

**Table 2:** Correlations among the *CNP* system and anthropometric parameters.

	Weight	BMI	Height
**CNP**	*p* = 0.02; R = 0.63	*p* = 0.02; R = 0.63	ns
**NPR-B**	*p* = 0.03; R = 0.60	*p* = 0.05; R = 0.54	*p* = 0.05; R = 0.54
**NPR-C**	*p* = 0.02; R = 0.62	*p* = 0.02; R = 0.40	ns

## Data Availability

The original contributions presented in this study are included in the article. Further inquiries can be directed to the corresponding author.

## Abbreviations

BrS	Brugada syndrome
ECG	Electrocardiogram
SCN5A	SCN5A-sodium voltage-gated channel alpha subunit 5
CNP	C-type natriuretic peptide
NPR-B	Natriuretic peptide receptor B or guanylate cyclase receptor B
NPR-C	Natriuretic peptide receptor C or clearance receptor
cGMP	Cyclic guanosine monophosphate
ddPCR	Droplet digital™ PCR
RNA	Ribonucleic acid
cDNA	Complementary desoxyribonucleic acid
